# Hereditary alpha tryptasemia: elevated tryptase, female sex, thyroid disorders, and anaphylaxis

**DOI:** 10.3389/falgy.2024.1461359

**Published:** 2024-11-12

**Authors:** Viktoria Puxkandl, Stefan Aigner, Wolfram Hoetzenecker, Sabine Altrichter

**Affiliations:** ^1^ Department for Dermatology and Venerology, Kepler University Hospital, Linz, Austria; ^2^ Center for Medical Research, Johannes Kepler University, Linz, Austria; ^3^ Institute of Allergology, Charité—Universitätsmedizin Berlin, Corporate Member of Freie Universität Berlin and Humboldt-Universität zu Berlin, Berlin, Germany; ^4^ Fraunhofer Institute for Translational Medicine and Pharmacology ITMP, Allergology and Immunology, Berlin, Germany

**Keywords:** hereditary alpha tryptasemia, HaT, elevated tryptase, female sex, thyroid disorder, thyroid gland, anaphylaxis, *TPSAB1*

## Abstract

**Introduction:**

The clinical significance of elevated baseline serum tryptase (BST) in the absence of mast cell disorders or allergic reactions has long been unclear. Recently, a genetic variation of the *TPSAB1* gene, which among others encodes for alpha tryptase, has been reported and named hereditary alpha tryptasemia (HaT). HaT has been linked to various manifestations, including severe allergic reactions. However, clinical studies are limited. In this study, we aimed to determine HaT prevalence and characterize its clinical manifestations in patients at a specialized allergy center.

**Methods:**

From January 2022 to December 2023, patients with elevated BST at least once were screened for HaT at the outpatient clinic. A control group included patients with a history of anaphylaxis undergoing specific Hymenoptera immunotherapy. *TPSAB1* copy numbers, BST levels, and clinical parameters were assessed and analyzed.

**Results:**

Of 47 patients with elevated BST (≥11.4 µg/L), 93% showed increased *TPSAB1* copy numbers. Individuals diagnosed with HaT displayed a BST range between 12.3 and 28.4 µg/L, with 84.1% associated with *TPSAB1* duplication and 15.9% with triplication. HaT predominated in women (86.4%) and was associated with thyroid disease (27.3%). Over half had a history of anaphylaxis (54.5%), which was mainly low-grade.

**Discussion:**

In patients with elevated BST but no mastocytosis, the most likely cause of elevated BST was an increase in the copy number of the *TPSAB1* gene. A heightened risk of anaphylaxis should be considered. Further research is needed to explore the predominance of women and the emerging link with thyroid disease.

## Graphical Abstract

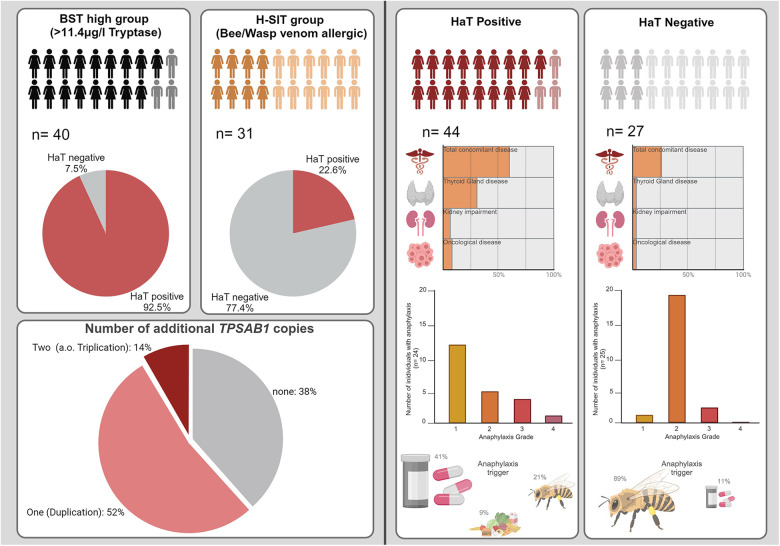

## 1 Introduction

Tryptase is a protein expressed mainly by mast cells and to a very minor extent by basophils, but no other cells. Persistently elevated baseline serum tryptase (BST) is an important screening tool for systemic clonal mast cell diseases, particularly systemic mastocytosis (1, 2), but can also be caused by other diseases such as renal insufficiency or other conditions (3, 4). Transiently elevated serum tryptase occurs immediately after mast cells are activated, for example, by an allergic reaction (5, 6).

Elevated BST in routine serological assays is often defined as >11.4 µg/L. However, an elevation of BST above this threshold appears to be quite common in the general population (7). Individuals with elevated BST are often screened for mastocytosis but then do not meet other criteria for the disease. Alternatively, an elevated BST may be a marker of hereditary alpha tryptasemia (HaT) (8, 9). This condition was first described in 2016 by Lyons et al. (10) and has since gained increasing attention due to the potential risk of multiple clinical manifestations, including a higher risk of life-threatening anaphylactic reactions (7). However, HaT and systemic mastocytosis can exist simultaneously and this is a risk factor for more severe anaphylactic reactions (9).

HaT is an autosomal dominantly inherited genetic trait that is estimated to affect 3%–5% of the Western population and is the most common cause of elevated BST in these individuals. HaT is caused by an increased germline *TPSAB1* copy number. *TPSAB1* is located together with *TPSB2* on chromosome 16p13.3. *TPSAB1* codes for alpha- and beta-tryptase isoforms, whereas *TPSB2* exclusively codes for beta-tryptase (11). The remaining isoforms in this locus, *TPSG1* and *TPSD1*, encode for tryptases that are not routinely measured in the standard assays (3). Bioactive tryptase tetramers derive exclusively from *TPSB2* (which encodes β-tryptase) and *TPSAB1* (which encodes α- or β-tryptase) (12). Tryptases derive from pre-tryptase, a 274-amino acid peptide, which is processed into pro-tryptase, a 257-amino acid peptide, and can also be processed into 245-amino acid mature tryptases, which are then stored in mast cell granules as tetramers and stabilized by heparin (13). While mature β-tryptase tetramers are primarily present in mast cells, cytoplasmatic granules are released following mast cell activation e.g., during allergic reactions (3). Heterotetramers are thought to have unique functional properties that can lead to the activation of mast cells and other cell types, some of which may underlie specific clinical manifestations of HaT (14). Pro-tryptases, which have not yet been converted into mature tetrameric structures, are secreted into the serum in their monomeric form and account for the majority of measured BST in healthy individuals in a steady state (3).

Most individuals with HaT have increased BST and may have associated multisystemic symptoms (e.g., cutaneous, gastrointestinal, neurological, and psychiatric symptoms and anaphylaxis). Some of these patients respond well to omalizumab (an anti-IgE antibody) or antihistamines (15). However, patients without clinical symptoms have also been described (11, 16). BST and presumably clinical symptom severity exhibit a gene–dose relationship with *TPSAB1*, with higher tryptase levels and greater symptom severity being correlated with increasing numbers of alpha-encoding *TPSAB1* copies (3). Overall, HaT encompasses a broad spectrum of BST and should be considered in patients with symptoms of mast cell activation and tryptase levels already greater than 6-8 µg/L (11, 16).

During mast cell degranulation, as occurs in IgE-mediated immediate hypersensitivity reactions, mature tryptases are released along with other mast cell mediators and contribute to the symptoms of type I allergic reactions. Serum tryptase is, therefore, a useful biomarker for the diagnosis of anaphylaxis in this context (3). Elevated tryptase levels can also be detected in patients with renal impairment and of older age (4, 17). In contrast, in other mast cell-activating diseases such as urticaria, only slight tryptase level elevations are detectable, usually not outside the reference values (18), but Robey et al. (11) reported a frequent history of urticaria and angioedema in people with a *TPSAB1* copy number variation.

There are currently only a limited number of published studies involving HaT patients. Recently, HaT has also been shown to be associated with a severe Hymenoptera venom allergy and clonal mast cell disease (8, 9). Some of these studies suggested an increased risk and severity of anaphylaxis in patients with HaT (19, 20), while others found no such correlation (21, 22).

To date, it is still a debate (23) and not completely clear which clinical symptoms, including allergic reactions/anaphylaxis, are associated with HaT, whether the symptoms differ according to the number of gene copies, and if different serological markers might be associated with them.

The aims of the study were to (i) determine the prevalence of HaT in patients with documented elevated baseline serum tryptase at least once, (ii) determine the prevalence and type of *TPSAB1* copy variation in this cohort, and (iii) evaluate the clinical phenotypes and severity of anaphylaxis in individuals with a *TPSAB1* variation. As a control group, we enrolled individuals with a Hymenoptera venom allergy/anaphylaxis undergoing Hymenoptera-specific immunotherapy (H-SIT) to represent a randomly selected group of individuals with anaphylaxis, regardless of their baseline serum tryptase.

## 2 Materials and methods

### 2.1 Patients

At the allergy outpatient clinic (Department of Dermatology and Venereology, Comprehensive Allergy Center, Kepler University Hospital), BST is a routine measurement for all patients admitted with symptoms fitting the immediate type [type I according to Coombs and Gel l (24)] of allergic or pseudoallergic reactions, urticaria, or suspected mastocytosis, or patients with elevated serum tryptase in external blood analysis. In this study, we retrospectively analyzed patients (age > 18 years) with an elevated BST at least once in these routine measurements with an excluded diagnosis of systemic mastocytosis [absence of a point mutation of the c-Kit gene (D816V) in the blood or negative bone marrow biopsy] (1, 2), who were screened for HaT between January 2022 and December 2023. These patients with an elevated BST at least once in the blood tests were included in the BST-high group.

In addition, patients who experienced anaphylaxis after a Hymenoptera sting and were undergoing Hymenoptera-specific immunotherapy (H-SIT group) were screened for HaT and mastocytosis (via blood test) and served as the control group, thereby representing a randomly selected group of patients with one or more confirmed anaphylactic events regardless of their BST.

Seven patients had both elevated BST and experienced anaphylaxis/underwent specific Hymenoptera immunotherapy. These patients were grouped into the H-SIT group since this was their primary reason for their admission to the allergy center. In total, 71 people were screened for HaT.

Ethical approval was obtained from the local ethics committee (ECS Nr. 1177/2023 Version 3). All the patient's records were pseudonymized in accordance with data protection and local ethics regulations.

### 2.2 Clinical assessments

Patient data, including age, sex, documented allergies, and comorbidities were obtained from the patient charts. The patient charts were screened for documented allergic comorbidities such as atopic dermatitis, allergic rhinitis, asthma, and type IV contact allergy, and/or mast cell-driven diseases such as acute or chronic urticaria. Documented anaphylactic reactions were categorized according to Ring and Messmer's (25) grading scale. The triggers were categorized into medications (antibiotics, local anesthesia, analgesics, and others), Hymenoptera venom, food, contrast agent, or unknown.

The patient charts were screened for comorbid diseases of special interest in association with elevated BST (4, 26), such as renal disease and oncological diagnoses. History of urticaria was also included, as it was previously described as an association in the literature (11). Patient files were further screened for concomitant diseases. They were included in a separate analysis if more than 10% of the patients presented with such a diagnosis.

### 2.3 Laboratory assessments

Each patient was screened for HaT using ethylenediaminetetraacetic acid (EDTA) whole blood samples at an external laboratory (MVZ Martinsried GmbH; https://www.medicover-diagnostics.de/lvz/panels/hereditaere-alpha-tryptasaemiehat–7826, last accessed 6 September 2024) via digital droplet PCR (ddPCR) from isolated and digested DNA samples with customized primers and probes for α- and β-tryptase, obtained from published sequences (20). HaT was diagnosed if there was evidence of a *TPSAB1* gene number variation, such as one or more additional gene copies (duplication or triplication). The different detected genotypes are shown in Supplementary Table S2, with the first letter before and after the colon referring to the *TPSB2* gene locus and the following letters to *TPSAB1*.

Total serum IgE and serum tryptase levels were assessed at the central nuclear laboratory of the clinic using the ImmunoCAP System® (Phadia Laboratory Systems, Thermo Fisher Scientific Inc., Uppsala, Sweden). Total IgE levels >112 IU/ml and tryptase levels >11.4 µg/L were considered as elevated.

Data from routine clinical assessments and laboratory values such as glomerular filtration rate (in-house laboratory) were included if available. Renal impairment was defined from patient records or with a documented glomerular filtration rate less than or equal to 60 ml/min/1.73 m^2^.

Thyroid gland disease was defined based on entries in patient records, the documented presence of elevated autoantibodies (anti-thyroid peroxidase or thyroglobulin antibodies), and/or a thyroid hormone substitution at the time of the examination.

### 2.4 Statistical analyses

Statistical analyses were performed using ISM SPSS Statistics (version 29.0.0.0).

A normal distribution was determined by significance (*p* < 0.05) in the Kolmogorov–Smirnov test. Statistical analysis was performed using the Mann–Whitney test if values were not normally distributed and a *t*-test if normally distributed for group comparisons. Results are shown as median with interquartile range (IQR) if not normally distributed and as mean (±standard deviation) if normally distributed. Binomial variables were analyzed using Fisher’s exact test. A *p*-value <0.05 was considered statistically significant.

## 3 Results

### 3.1 Of the patients with elevated BST, 93% present with an increase in *TPSAB1* copy number

In this study, we screened 40 patients with elevated BST for their *TPSAB1* copy number. These patients showed tryptase levels between 11.4 and 28.4 µg/L. Almost all (93%) of our recruited patients with elevated BST had a detectable increase in *TPSAB1* copy number (see Table 1).

**Table 1:** Baseline characteristics of the BST-high and H-SIT patient cohorts.

	BST-high *n* = 40	H-SIT *n* = 31	*p*-value
Serum tryptase, median (IQR) (µg/L)	16.1 (6.2)	6.03 (6.5)	0.004**
Female sex, *n* (%)	34 (85.0)	12 (38.7)	<0.001**
Age, median (IQR) (years)	60.5 (18)	49 (20)	0.006**
HaT, *n* (% pos)	37 (92.5)	7 (22.6)	<0.001**

BST, baseline serum tryptase; H-SIT, Hymenoptera-specific immunotherapy; IQR, interquartile range; HaT, hereditary alpha tryptasemia; pos, positive.

Statistical analysis was performed using the Mann–Whitney test (the Kolmogorov–Smirnov test rejects a normal distribution) and Fisher’s exact test.

Significant results are indicated with asterisks: **p < 0.01.

**Figure 1:**
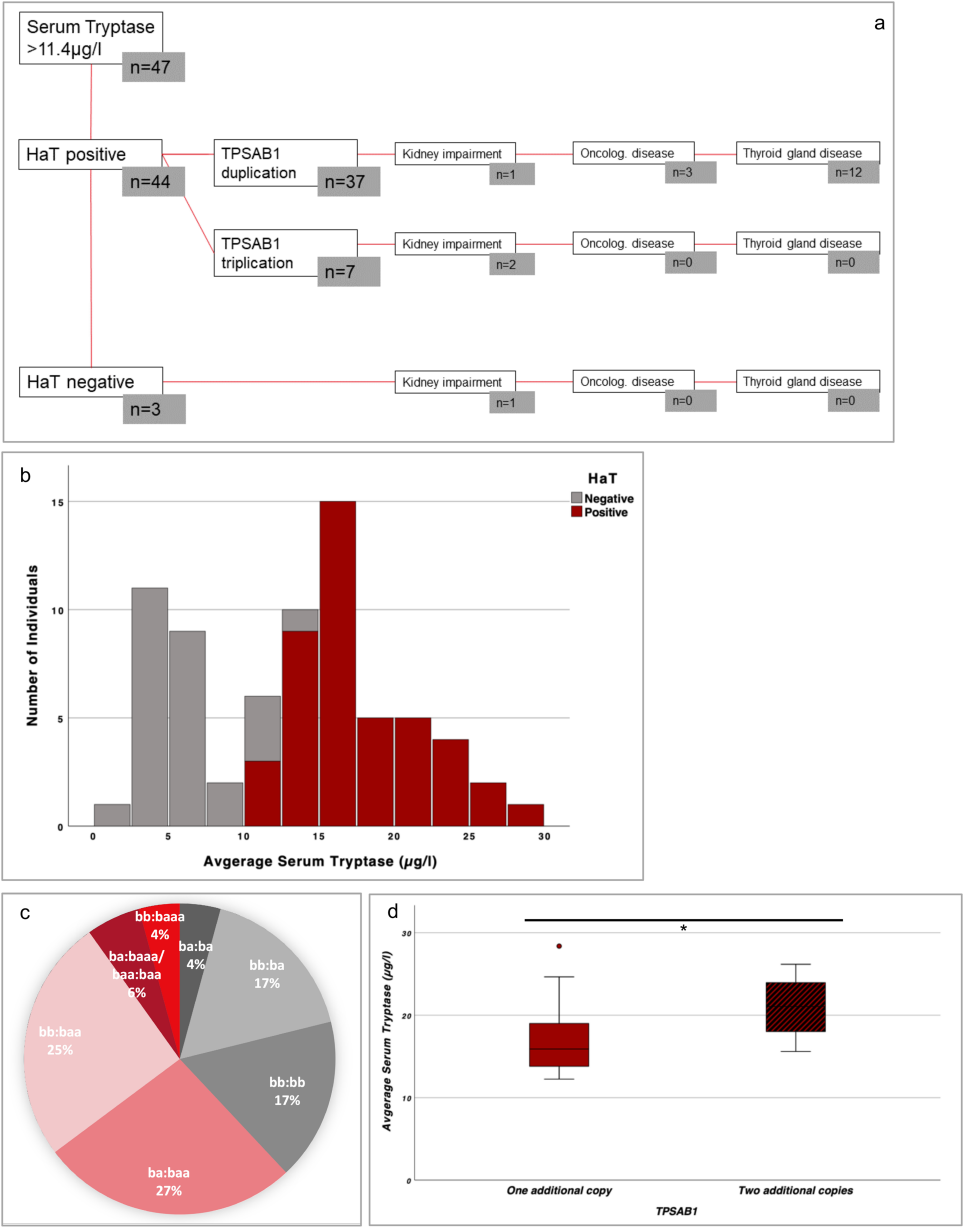
**(a)** Clinical characteristics of people with BST >11.4 µg/L (including seven patients from the H-SIT cohort). **(b)** Average serum tryptase of the cohort as a stacked bar graph. HaT is indicated in red. **(c)** HaT genotype prevalence. Physiological variations are gray, one additional *TPSAB1* copy is light red, and two additional *TPSAB1* copies are dark red. **(d)** Boxplot comparing mean BST levels with one or two additional *TPSAB1* copies. The bold line represents the median, the boxes the lower and upper quartiles (Q1/Q3), and the whiskers the minimum/maximum (except for one outlier—marked as a circle). Statistical analysis was performed using the Mann–Whitney test (the Kolmogorov–Smirnov test rejects a normal distribution). The group with one additional copy is indicated in red; the group with two additional copies is indicated in red with black stripes. Significance (*p* = 0.017) is indicated with one asterisk. HaT, hereditary alpha tryptasemia; Oncolog, oncological.

When considering all patients with elevated BST, HaT was not diagnosed in only three patients. In one of these patients, reduced renal function could possibly account for the elevated tryptase level. In the other two patients, no clear explanation for the elevated BST has been found to date (see Figure 1a and Supplementary Table S1). However, those patients were significantly older compared to those with a detected increase in *TPSAB1* copy number (*p* = 0.05).

Further, our second cohort of 31 patients with diagnosed Hymenoptera anaphylaxis who underwent specific immunotherapy were all screened for HaT. Seven patients (22.6%) in this cohort presented with elevated serum tryptase levels (median 19 µg/L; IQR 5.8) and all of these patients had a detectable increase in *TPSAB1* copy number (see Table 1). Of these, six were female and one person was male.

Notably, 86.5% of the BST-high cohort but only 38.7% of the H-SIT cohort were female (see Table 1).

From both cohorts, patients with a HaT diagnosis had a BST range between 12.3 and 28.4 µg/L (see Figure 1b). Their median BST (16.4 µg/L; IQR 6.4) was significantly higher compared to the median BST in the non-HaT patient group (5.5 µg/L; IQR 3.28; *p* < 0.001) (see Supplementary Figure S1 and Table S2).

### 3.2 Most HaT patients had a *TPSAB1* duplication, whereas a triplication was rare but associated with higher BST

One additional copy (duplication) of TPSAB1 was more frequent than two additional copies (i.e., triplication). The most frequent genotype in people with HaT was ba:baa (see Figure 1c and Supplementary Table S2*)*.

HaT patients with two additional *TPSAB1* copies (*n* = 7) showed a significantly higher BST (median 21.6 µg/L; IQR 9.1; range 15.6–26.2 µg/L) compared to patients with duplication (*n* = 37; median BST 15.9 µg/L; IQR 5.6; range 12.3–28.4 µg/L; *p* = 0.020) (see Figure 1d).

### 3.3 Hat diagnosis was associated with female sex and thyroid gland disease

When HaT patients and the persons without increased *TPSAB1* copy number were analyzed regarding clinical characteristics, we noticed that significantly more women than men were in the HaT patient cohort (see Table 2).

**Table 2:** HaT cohort characteristics.

		HaT-positive (*n* = 44)	HaT-negative (*n* = 27)	*p*-value
Serum tryptase, median (IQR) (µg/L)		16.4 (6.4)	5.5 (3.3)	<0.001**
Female sex, *n* (%)		38 (86.4)	8 (29.6)	<0.001**
Age, median (IQR) (years)		60 (22)	51 (26)	0.234
Total concomitant disease, *n* (% pos)		26 (59.1)	7 (25.9)	0.008**
Kidney impairment, *n* (% pos)		3 (6.8)	1 (3.7)	1.00
Oncological disease, *n* (% pos)		3 (7.0)	1 (3.7)	0.619
Acute urticaria, *n* (% pos)		3 (6.8)	1 (3.7)	1.00
CSU, *n* (% pos)		9 (20.5)	1 (3.7)	0.077
Thyroid gland disease, *n* (% pos)	Total	12 (27.3)	1 (3.7)	0.013*
	Nodular goiter	5 (11.4)	0 (0)	0.149
	Hashimoto’s thyroiditis	4 (9.1)	0 (0)	0.290
	Thyroid autoantibodies	5 (11.4)	0 (0)	0.149
	Thyroid substitution therapy	8 (18.2)	1 (3.7)	0.139

CSU, chronic spontaneous urticaria; IQR, interquartile range; HaT, hereditary alpha tryptasemia; pos, positive.

Statistical analysis was performed using the Mann–Whitney test (the Kolmogorov–Smirnov test rejects a normal distribution) and Fisher’s exact test.

Significant results are indicated with an asterisk: *p < 0.05; **p < 0.01.

HaT patients were significantly more likely to have non-allergic comorbidities, often thyroid disease (see Table 2). A total of 12 people with HaT were found to have an abnormality of the thyroid gland. Of these, nine had a documented thyroid disease. The remaining three people had either documented thyroid autoantibodies or were taking a specific substitution medication without a documented/recorded underlying disease. The most common thyroid diseases were nodular goiter (41.6%) and Hashimoto's thyroiditis (33.3%). In 5 of the 12 patients, thyroid autoantibodies were documented. Approximately two-thirds of the patients with a thyroid diagnosis and diagnosis of HaT required thyroid hormone substitution at the time of assessment.

Because of the female predominance in the HaT cohort, we separately compared the female and male patient cohorts (see Supplementary Tables S3A,B) to the patients without HaT, which revealed that these differences were predominantly visible in the male, rather than in the female population.

There were no significant differences when comparing patients with duplication and those with two additional *TPSAB1* copies/triplication (see Supplementary Table S4).

### 3.4 HaT diagnosis was associated with low-grade anaphylaxis

In our non-HaT group (*n* = 27), 25 (92.6%) patients had a history of anaphylaxis, most of them (*n* = 24; 88.9%) following Hymenoptera stings (see Table 3). In contrast, more than half (54.5%) of the patients with HaT displayed a history of anaphylaxis. HaT was associated with low-grade anaphylaxis (27.3% grade I), whereas the non-HaT group had the highest incidence of grade II (70.4%) reactions. Of note, among all analyzed cases, there was only one patient with grade IV anaphylaxis (after acetylsalicylic acid intake) and this specific patient also had HaT. Medication-triggered reactions (40.9%) and more specific analgesics [non-steroidal anti-inflammatory drugs (NSAIDs); 25%] were the most common trigger of reactions of HaT patients. A history of anaphylaxis was rare in the male patients with HaT (16.7%) compared to women with HaT (60.5%) (see Supplementary Tables S5A,B).

**Table 3:** Anaphylaxis characteristics (HaT-positive vs. HaT-negative).

		HaT-positive (*n* = 44)	HaT-negative (*n* = 27)	*p*-value
Total IgE, median (IQR) (U/ml)		50.8 (153.6)	60 (126.5)	0.503
AR *n* (% pos)		13 (29.5)	2 (7.4)	0.036*
History of anaphylaxis, *n* (% pos)^a^		24 (54.5)	25 (92.6)	<0.001*
Grade, *n* (% pos)	Anaphylaxis grade I	12 (27.3)	1 (3.7)	0.013*
	Anaphylaxis grade II	5 (11.4)	20 (74.1)	<0.001**
	Anaphylaxis grade III	4 (9.1)	2 (7.4)	1.00
	Anaphylaxis grade IV	1 (2.3)	0 (0)	1.00
Culprit, *n* (% pos)	Hymenoptera	9 (20.5)	24 (88.9)	<0.001**
	Food	4 (9.1)	0 (0)	0.290
	Total medications	18 (40.9)	3 (11.1)	0.008**
	• Antibiotics	3 (6.8)	2 (7.4)	1.00
	• NSAIDs (including intolerance)	11 (25)	1 (3.7)	0.023*
	• Local anesthetics	2 (4.5)	0 (0)	0.522
	• Other	6 (13.6)	3 (11.1)	1.00
	Contrast agent (CT or MRT)	2 (4.5)	0 (0)	0.522
	Unknown	1 (2.3)	0 (0)	1.00
Type IV sensitizations, *n* (% pos)		4 (9.1)	2 (7.4)	0.679

IQR, interquartile range; HaT, hereditary alpha tryptasemia; pos, positive; AR, allergic rhinitis; NSAIDs, non-steroidal anti-inflammatory drugs; MRT, magnetic resonance tomography; CT, computertomographie.

Statistical analysis was performed using the Mann–Whitney test (the Kolmogorov–Smirnov test rejects a normal distribution) and Fisher’s exact test. Anaphylaxis was graded according to Ring and Messmer (25).

^a^ Grade of anaphylaxis was not available for two individuals in the HaT-positive group and four in the HaT-negative group.

Significant results are indicated with an asterisk: *p < 0.05; **p < 0.01.

In the Hymenoptera venom-allergic cohort, grades I and III anaphylaxis were more common in patients with HaT (33.3% and 22.2%, respectively), compared to the HaT-negative patients who mainly reacted with grade II (79.2%), see Table 4.

**Table 4:** Patients with reported anaphylaxis after a Hymenoptera venom sting and their grouping into HaT-positive/negative subgroups.

		Anaphylaxis after a Hymenoptera venom sting		
		HaT-positive (*n* = 9)	HaT-negative (*n* = 24)	*p*-value
Female sex, *n* (%)		8 (88.8)	6 (25)	0.002**
Grade, *n* (% pos)^a^	Anaphylaxis grade I	4 (44.4)	1 (4.2)	0.013*
	Anaphylaxis grade II	3 (33.3)	20 (83.3)	0.033*
	Anaphylaxis grade III	2 (22.2)	2 (8.3)	0.295
	Anaphylaxis grade IV	0 (0)	0 (0)	^a^
Culprit, *n* (% pos)	Bee	2 (22.2)	9 (37.5)	0.681
	Wasp	4 (44.4)	13 (54.2)	0.708
	Bee and wasp	3 (33.3)	2 (8.3)	0.111

Statistical analysis was performed using Fisher’s exact test. Anaphylaxis was graded according to Ring and Messmer (25). HaT, hereditary alpha tryptasemia; pos, positive.

^a^ Grade of anaphylaxis was not available for one individual in the HaT-negative group.

Significant results are indicated with an asterisk: *p < 0.05; **p < 0.01.

Two additional *TPSAB1* copies did not increase the frequency or severity of the anaphylactic reactions (see Supplementary Table S6). Only two of seven (28.6%) patients with two additional gene copies had a history of anaphylaxis, compared to 59.5% in the cohort with only one additional copy. The reactions in the two patients in the group with two additional copies were grades II and III.

## 4 Discussion and limitations

The results from our study support that elevated BST levels in the absence of evidence of mastocytosis are in most cases associated with an increased *TPSAB1* copy number. A previous study of patients with moderate elevations in BST in a regional health system in the United States (US) reported lower rates of HaT (63.8%) but higher rates of chronic kidney disease (12.1%) and clonal myeloid disorders (20.7%) (27). Differences in numbers are possibly due to preselection of our patients, who were already admitted to our allergy center, and due to the different BST cut-off compared to the US study (≥7.5 µg/L).

In our study, only three individuals with elevated BST (>11.4 µg/L) did not have increased *TPSAB1* copy number. Interestingly, these three individuals were significantly older compared to the rest of the cohort. Vos et al. (17) showed that people of older age and with a body mass index (BMI) >25 kg/m^2^ tend to have greater tryptase values. Only one of the three patients had a reported kidney disease (see Supplementary Table S1), which can be associated with elevated tryptase levels (4). The BMI of our study cohort was not assessed in our study but should be considered in further projects. Further, a history of urticaria could also be an influencing factor for increased tryptase (18) in individuals with elevated BST without HaT or kidney dysfunction, as documented in two of our three cases.

In our cohort with validated anaphylaxis to Hymenoptera, more than 20% had detectable HaT and elevated serum tryptase. This rate was higher than the expected HaT prevalence of 5% in the average Western population (11) and hints toward a higher rate of Hymenoptera anaphylaxis in HaT patients.

Among our HaT-positive patients, their BST ranged between 12.3 and 28.4 µg/L. However, HaT can also be present in patients with a BST below the common lab threshold (11.4 µg/L) as previously described (11). Lower thresholds lead to an increased HaT detection rate but will also include a higher range of individuals without HaT (27). This issue emphasizes the need for a re-evaluation of the commonly used tryptase threshold in day-to-day applications.

Most of our HaT patients had a *TPSAB1* duplication and two additional copies/triplication were only observed in approximately 15% of the patients. These numbers are in line with previous reports from other cohorts (11). The gene dosage effect on BST was also found in our cohort (3). Significantly higher tryptase levels were seen in patients with triplicates, with BST levels above 20 µg/L.

As HaT is an autosomal dominant genetic trait, an equal distribution between men and women should be assumed. Nevertheless, we were able to observe a strong female predominance in our patients. The previously mentioned study on 109 subjects with basal tryptase values of 7.5 ng/ml or greater (27) and other studies (19, 21) did not observe such a correlation. However, several studies reported a female predominance (16, 28), with more than two-thirds of the patients with HaT being women, which is consistent with the results seen in our cohort. This female predominance could be reinforced by a selection bias or, as speculated by Giannetti et al. (16), be due to a connection with sex hormones. This study and the study by Giannetti et al. (16) showed a median age of >50 years in the female cohort, which is why the menopause could be hypothesized to have an influence. However, data supporting any of these theories are missing.

In the Hymenoptera anaphylaxis group, fewer women than men were included. Still, in this cohort, HaT was associated with the female sex (six women out of a total of seven HaT-positive patients), again emphasizing the female predominance. In general, previous studies have shown an increased prevalence of women in drug allergies (29), although gender predominance is controversial in studies on other allergy culprits such as Hymenoptera venom (30).

In our HaT patient cohort, we detected significantly more non-allergic comorbidities, most strikingly thyroid diseases. To our knowledge, an association with thyroid disease was only reported in one case to date (31). Since we had a high rate of women in our cohort and women tend to have more often thyroid diseases (32, 33), we compared women and men separately. In that analysis, we still observed the same association with thyroid diseases in approximately 26% of women with HaT and 33% of men with HaT vs. only 12.5% and 0% in the respective HaT-negative cohort (see Supplementary Tables 3A,B). Possibly due to the low patient numbers in the same-sex analysis, these differences were only significant in the male cohort. Interestingly, there have been reports of the possible involvement of mast cells in auto-antigen presentation in the progression of autoimmune thyroid disease (34) as well as a possible contribution of mast cells in thyroid carcinoma growth (35), making this area of interest for further studies. A more precise survey of the diagnosis of thyroid disease would be desirable for future studies, with prospective data collection and precise classification. Due to the retrospective model in this study and the fact that the correlation between HaT and thyroid disease only became apparent during the data analysis, further information on thyroid disease may have been missed. However, other comorbidities were comparable between HaT-positive and HaT-negative patients.

Approximately half of our HaT patient cohort had a clinical history of a reaction that was classified as an anaphylactic reaction. Half of these reactions (12 of 24) were classified as grade I, approximately 20% (5 of 12) as grade II, and only 5 and 1 of the 24 as grades III and IV, respectively (see Table 3). grade I reactions are localized to the skin only and include reactions such as skin erythema, itch, wheals, and/or angioedema. All these features are also present in patients with urticaria and can be misdiagnosed, especially with acute urticaria. Urticaria was reported to be associated with HaT by Robey et al. (11) in more than 50% of their cases. We also observed higher rates of documented acute and chronic urticaria in the patient files for the HaT-positive patients which were about double as much compared to the HaT-negative patients, but these differences were not statistically significant. As our patients had not been specifically screened for their urticaria history, cases of acute urticaria may have been missed/underreported or maybe even misinterpreted as grade I anaphylaxis. Furthermore, urticaria is associated with thyroid disease (36, 37), which we also noticed in our HaT cohort, and female sex (38). It has been shown that some patients with chronic spontaneous urticaria (CSU) express IgE anti-thyroid peroxidase antibodies which may lead to an autoallergic activation of mast cells and thereby inducing CSU (39), however, another research group showed no such correlation (40).

As summarized in a recent review (23), several studies have shown that elevated serum tryptase and HaT are associated with a higher risk of severe anaphylaxis (7, 16, 29), whereas other studies did not show such a correlation (21, 22). In our cohort, this genetic variation was associated with a greater than 50% chance of having experienced an anaphylactic reaction in the past but not, however, with severe anaphylaxis. Only approximately 10% (6 of 44 HaT patients) had a grade III or IV reaction. This was comparable to the non-HaT group (7.4% grade III, no grade IV) which mostly consisted of Hymenoptera allergic patients. The most common trigger for anaphylactoid reactions was NSAIDs in our cohort. Interestingly, an association between HaT and NSAID intolerance has not been described before. In previous publications, elevated baseline tryptase was rare in NSAID-intolerant patients (42) and most patients with clonal mast cell disease tolerate NSAIDs (43). However, NSAIDs are a typical trigger for acute urticaria/grade I reactions. However, it has been previously described that patients with an increased *TPSAB1* copy number are more likely to present with cutaneous symptoms, such as flushing or itching (11, 21). Therefore, it can be hypothesized that the patients referred to our comprehensive allergy center due to a history of low-grade anaphylaxis have experienced symptoms of mast cell activation and thereby eventually HaT, even though they were missing the involvement of another organ. Further investigation with a structured medical history and an even larger study cohort would be necessary to better decipher the trigger for the reactions in these patients. Therefore, easily accessible tests would also be needed, as recently described (44).

Hymenoptera venom allergy and more severe reactions have been described to be associated with HaT (8). We also detected HaT in a high number of Hymenoptera venom-allergic patients (24%) undergoing H-SIT. Within the H-SIT cohort, HaT was associated with significantly more frequent low-grade reactions and also with slightly but not significantly higher numbers of grade III reactions, compared to the non-HaT H-SIT patients. The latter group mainly presented with grade II anaphylaxis, but higher-grade reactions were rare overall in both groups. However, gene triplication did not increase the risk of more severe reactions in our studied cohort even though we saw a gene dosage effect on BST. However, in the literature, elevated BST has been linked multiple times with a higher risk of severe anaphylaxis (41, 45).

Overall, in our study, we have the limitations of a restricted number of patients, a possible referral bias as a tertiary allergy center, and a retrospective study design with possibly incomplete medical histories and missing data. In addition, a selection bias cannot be excluded, as patients with a one-time elevated tryptase, with a relatively high threshold value (11.4 µg/L), were selected and persons with an allergic history were used as a comparison group. However, we were able to confirm previous reports, but also found differences in terms of female predominance and reported for the first time an association with thyroid gland disease. A history of anaphylaxis was prevalent in the HaT patients but mostly of a lower grade, except for Hymenoptera allergy, where patients had a higher risk of more severe reactions. Of note, this study only included one patient with grade IV anaphylaxis (after acetylsalicylic acid).

There is a need for additional comprehensive research regarding the association of HaT and thyroid gland diseases, as well as the predominance of the female sex.

## 5 Conclusion

In conclusion, the findings underline the role of *TPSAB1* variations, particularly the prevalence of a *TPSAB1* copy number variance in patients with elevated BST levels. The wide range of BST levels observed in patients diagnosed with HaT highlights the heterogeneity of this condition. Furthermore, the association of *TPSAB1* triplication with elevated BST levels supports the correlation between gene dosage and tryptase expression.

The significant association of HaT diagnosis with female sex and thyroid gland disease highlights the importance of considering these factors in clinical assessment and management. The higher prevalence of low-grade anaphylaxis in HaT patients also emphasizes the clinical implications of this condition. These findings collectively contribute to our understanding of the complex interplay between genetic predisposition, tryptase levels, and clinical phenotype in HaT, establishing the foundation for future diagnostic strategies.

## Data Availability

The datasets used and/or analyzed during this study are available from the corresponding author on reasonable request.
