# Supplementary material for: Hereditary alpha tryptasemia: elevated tryptase, female sex, thyroid disorders, and anaphylaxis
Source: Front Allergy. 2024 Nov 12;5:1461359. doi: 10.3389/falgy.2024.1461359 (PMC11588693; doi:10.3389/falgy.2024.1461359)
Supplement: Supplementary file 1 [file Datasheet1.docx]

**Hereditary alpha Tryptasemia: Elevated Tryptase, Female Sex, Thyroid Disorders and Anaphylaxis**

Viktoria Puxkandl^1,2^, Stefan Aigner^2^, Wolfram Hoetzenecker^1,2^, Sabine Altrichter^1,2,3,4^

1. Department for Dermatology and Venerology, Kepler University Hospital, Linz, Austria
2. Center for medical research, Johannes Kepler University, Linz, Austria
3. Institute of Allergology, Charité – Universitätsmedizin Berlin, corporate member of Freie Universität Berlin, Humboldt-Universität zu Berlin, and Berlin Institute of Health, Berlin, Germany;
4. Fraunhofer Institute for Translational Medicine and Pharmacology ITMP, Allergology and Immunology, Berlin, Germany

## **Supplementary Figures and Tables**


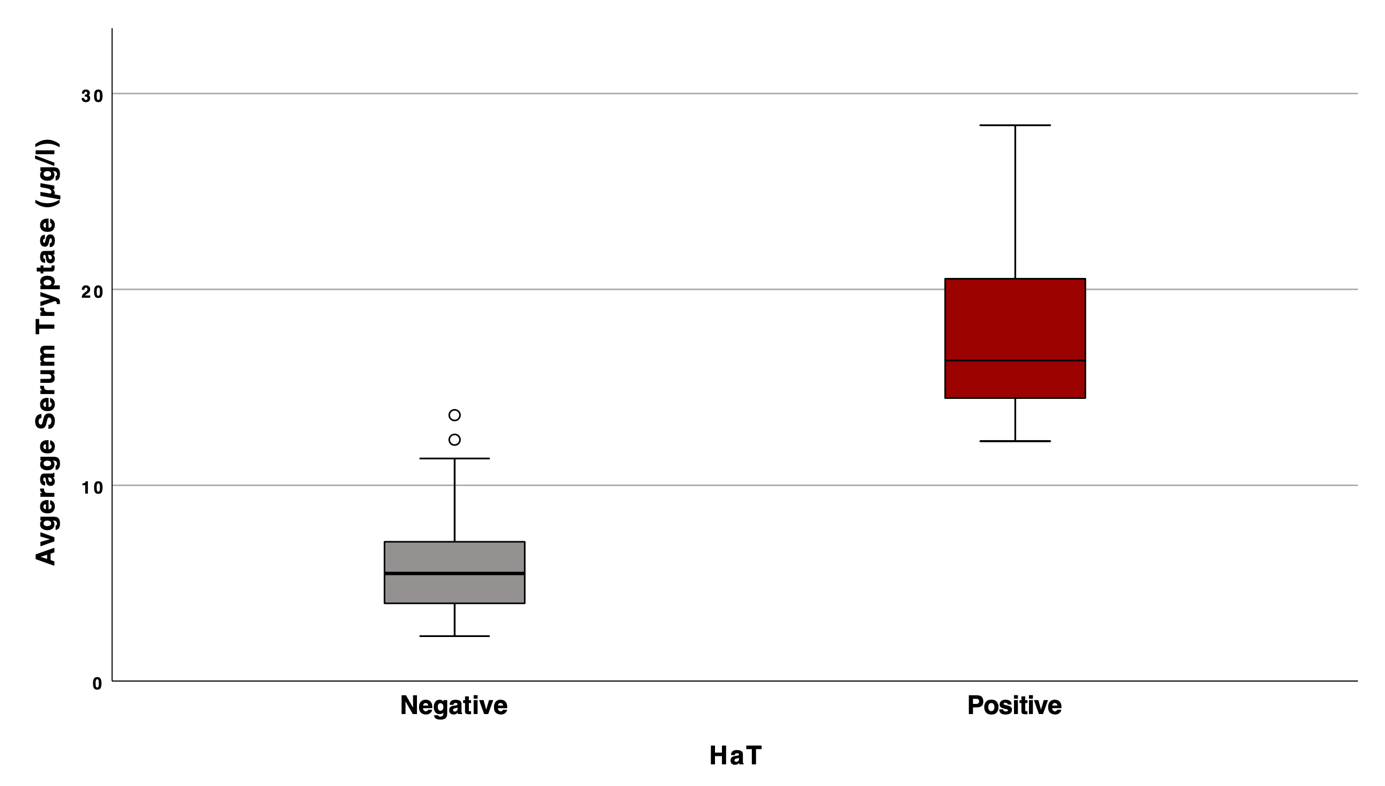


**

Supplementary Figure 1 - Boxplot comparing Average BST levels with positive and negative TPSAB1- copy number increase.
Bold line represents median, the boxes lower and upper quartile (Q1/Q3) and whiskers minimum/maximum (except for two outliers - marked as a circle).. Statistical analysis was performed using a Mann-Whitney-Test (Kolmogorov-Smirnov rejects normal distribution). HaT indicated in red. Significance (p<0.01) is indicated with two asterisks **.
Abbreviations: HaT – Hereditary alpha Tryptasemia.

|  | Total | 1 | 2 | 3 |
| --- | --- | --- | --- | --- |
| Average Serum Tryptase  (mean (SD), µg/l) | 12.4 (1.1) | 12.3 | 13.6 | 11.4 |
| Female Sex (n; %) | 2 (66.7) | No | Yes | Yes |
| Age (mean (SD); years) | 73 (7.0) | 73 | 80 | 66 |
| HaT (n; % pos) | 0 | - | - | - |
| Kidney disease | 1 | - | 1 | - |
| Atopic Dermatitis | 1 | 1 | - | - |
| Acute Urticaria (+/- Angioedema) | 1 | - | - | 1 |
| CSU | 1 | 1 | - | - |
| History of anaphylaxis  (Grade not known) | 1 | - | 1 | - |
| AR | 1 | - | 1 | - |

Supplementary Table 1 - Characteristics of Patients without HaT but elevated BST
Abbreviations: SD – standard deviation, pos – positive, CSU – chronic spontaneous urticaria, AR – allergic rhinitis .

| *TPSAB1*  copy numbers | Genotype | n (%) | BST (median (IQR); µg/l) |
| --- | --- | --- | --- |
| none | ba:ba | 3 (4.2) | 4.6 (-) |
|  | bb:ba | 12 (16.9) | 5.7 (4.9) |
|  | bb:bb | 12 (16.9) | 5.8 (3.5) |
| 1 additional copy *(duplication)* | ba:baa | 19 (26.8) | 16.4 (5.2) |
|  | bb:baa | 18 (25.4) | 15.2 (4.9) |
| 2 additional copies | ba:baaa/ baa:baa | 4 (5.6) | 24 (4.5) |
|  | bb:baaa | 3 (4.2) | 16.3 (-) |
|  | Total | 71 (100) | 13.7 (10.7) |

Supplementary Table 2 - HaT Genotype Prevalence and mean BST level
Physiological gene-variation (TPSB2 and TPSAB1): TPSB2 exclusively codes for beta-tryptase (first b before and after colon). TPSAB1 codes for alpha- and beta-tryptase isoforms (subsequent letters a or b, respectively), TPSAB1 Duplication (equals 1 additional copy) and 2 additional copies or Triplication variation and the respective mean serum tryptase levels are given. Genotypes ba:baaa and baa:baa were grouped.
Abbreviations: BST – baseline serum tryptase, SD – standard deviation.

|  | | | | | |  |  | |  |  |
| --- | --- | --- | --- | --- | --- | --- | --- | --- | --- | --- |
|  | |  | HaT Positive  n=38 | | | | | HaT Negative  n=8 | p= |  |
| Serum Tryptase (mean (SD), µg/l) |  |  | 17.82 (4.36) | | | | | 7.28 (3.8) | <0.001** |  |
| Age (median (IQR), y) |  |  | 60 (17) | | | | | 61 (29) | 0.522 |  |
| Acute urticaria (n; % pos) |  |  | 3 (7.9) | | | | | 1 (12.5) | 0.548 |  |
| CSU (n; % pos) |  |  | 7 (18.4) | | | | | 0 (0) | 0.325 |  |
| Total concomitant disease (n; % pos) |  |  | 21 (55.3) | | | | | 3 (37.5) | 0.451 |  |
| Thyroid Gland disease (n; % pos) | total |  | 10 (26.3) | | | | | 1 (12.5) | 0.658 |  |
|  | Nodular goiter | | |  | 5 (13.2) | | | 0 (0) | 0.569 |  |
|  | Hashimoto-Thyroiditis | | |  | 3 (7.9) | | | 0 (0) | 1.00 |  |
|  | Thyroid Autoantibodies | | |  | 4 (10.5) | | | 0 (0) | 1.00 |  |
|  | Thyroid substitution therapy | | |  | 7 (18.4) | | | 1 (12.5) | 1.00 |  |
| Oncological disease (n; % pos) |  |  | 2 (5.3) | | | | | 0 (0) | 1.00 |  |
| Kidney impairment (n; % pos) |  |  | 2 (5.3) | | | | | 1 (12.5) | 0.444 |  |

Supplementary Table 3a – Female HaT Cohort Characteristics
Statistical analysis was performed using a t-test (Tryptase) and Mann-Whitney-Test (Age; as Kolmogorov-Smirnov rejects normal distribution) and fisher exact. Significant results were indicated with an asterisk (p<0.05*, p<0.01**).
Abbreviations: CSU – chronic spontaneous urticaria, SD – standard deviation, IQR – interquartile ranage, HaT – hereditary alpha tryptasemia, pos – positive, y – years.

|  | | | | |  |  | |  |  |
| --- | --- | --- | --- | --- | --- | --- | --- | --- | --- |
|  | |  | HaT Positive  n=6 | | | | HaT Negative  n=19 | p= |  |
| Serum Tryptase (median (IQR), µg/l) |  |  | 15.79 (3.2) | | | | 5.4 (3.1) | <0.001** |  |
| Age (mean (SD), y) |  |  | 52 (21.3) | | | | 48.79 (15) | 0.683 |  |
| Acute urticaria (n; % pos) |  |  | 0 (0) | | | | 0 (0) | # |  |
| CSU (n; % pos) |  |  | 2 (33.3) | | | | 1 (5.3) | 0.133 |  |
| Total concomitant disease (n; % pos) |  |  | 5 (83.3) | | | | 4 (21.1) | 0.012* |  |
| Thyroid Gland disease (n; % pos) | total |  | 2 (33.3) | | | | 0 (0) | 0.050* |  |
|  | Nodular goiter | |  | 0 (0) | | | 0 (0) | # |  |
|  | Hashimoto’s Thyroiditis | |  | 1 (16.7) | | | 0 (0) | 0.240 |  |
|  | Thyroid Autoantibodies | |  | 1 (16.7) | | | 0 (0) | 0.240 |  |
|  | Thyroid substitution therapy | |  | 1 (16.7) | | | 0 (0) | 0.240 |  |
| Oncological disease (n; % pos) |  |  | 1 (16.7) | | | | 0 (0) | 0.240 |  |
| Kidney impairment (n; % pos) |  |  | 1 (16.7) | | | | 0 (0) | 0.240 |  |

Supplementary Table 3b – Male HaT Cohort Characteristics
Statistical analysis was performed using a t-test (Age) and Mann-Whitney-Test (Tryptase; Kolmogorov-Smirnov rejects normal distribution) and fisher exact. Significant results were indicated with an asterisk (p<0.05*, p<0.01**).
Abbreviations: CSU – chronic spontaneous urticaria, SD – standard deviation, IQR – interquartile range, HaT – hereditary alpha tryptasemia, pos – positive, y – years.

|  | | | | | |  | ***TPSAB1 copy number (HaT)*** | |  |  |
| --- | --- | --- | --- | --- | --- | --- | --- | --- | --- | --- |
|  | |  | One additional copy/ Duplication  n=37 | | | | | Two additional copies (a.o. Triplication)  n=7 | p= |  |
| Serum Tryptase (median (IQR), µg/l) |  |  | 15.9 (5.6) | | | | | 21.6 (9.1) | 0.020* |  |
| Age (median (IQR), y) |  |  | 60 (21) | | | | | 60 (34) | 0.748 |  |
| Female Sex (n; %) |  |  | 32 (86.5) | | | | | 6 (85.7) | 1.00 |  |
| Acute urticarial (n; % pos) |  |  | 2 (5.4) | | | | | 1 (14.3) | 0.413 |  |
| CSU (n; % pos) |  |  | 8 (21.6) | | | | | 1 (14.3) | 1.00 |  |
| Total concomitant disease (n; % pos) |  |  | 24 (64.9) | | | | | 2 (28.6) | 0.103 |  |
| Thyroid Gland disease (n; % pos) | total |  | 12 (32.4) | | | | | 0 (0) | 0.163 |  |
|  | Nodular goiter | | |  | 5 (13.5) | | | 0 (0) | 0.574 |  |
|  | Hashimoto’s Thyroiditis | | |  | 4 (10.8) | | | 0 (0) | 1.00 |  |
|  | Thyroid Autoantibodies | | |  | 5 (13.5) | | | 0 (0) | 0.574 |  |
|  | Thyroid substitution therapy | | |  | 8 (21.6) | | | 0 (0) | 0.318 |  |
| Oncological disease (n; % pos) |  |  | 3 (8.1) | | | | | 0 (0) | 1.00 |  |
| Kidney impairment (n; % pos) |  |  | 1 (2.7) | | | | | 2 (28.6) | 0.061 |  |

Supplementary Table 4 - HaT Cohort Characteristics (TPSAB1 Duplication vs. Triplication)
Statistical analysis was performed using a Mann-Whitney-Test (Kolmogorov-Smirnov rejects normal distribution) and fisher exact. Significant results were indicated with an asterisk (p<0.05*, p<0.01**).
Abbreviations: a.o. – among others, CSU – chronic spontaneous urticaria, IQR – interquartile range, HaT – hereditary alpha tryptasemia, pos – positive, y – years.

|  |  |  | | |  |
| --- | --- | --- | --- | --- | --- |
|  |  | HaT Positive  n=38 | HaT Negative  n=8 | p = |  |
| Total IgE (median (IQR; U/ml) |  | 47.3 (124.1) | 61 (119.1) | 0.659 |  |
| AR (n; % pos) |  | 11 (28.9) | 1 (12.5) | 0.660 |  |
| History of anaphylaxis (n; % pos) (*) |  | 23 (60.5) | 7 (87.5) | 0.230 |  |
| Grade (n; % pos) | Anaphylaxis Grade I | 11 (28.9) | 1 (12.5) | 0.660 |  |
|  | Anaphylaxis Grade II | 5 (13.2) | 5 (62.5) | 0.007** |  |
|  | Anaphylaxis Grade III | 4 (10.5) | 0 (0) | 1.00 |  |
|  | Anaphylaxis Grade IV | 1 (2.7) | 0 (0) | 1.00 |  |
| Culprit (n; % pos) | Hymenoptera | 8 (21.1) | 6 (75) | 0.006** |  |
|  | Food | 4 (10.5) | 0 (0) | 1.00 |  |
|  | Medications total | 17 (44.7) | 2 (25) | 0.440 |  |
|  | - Antibiotics | 3 (7.9) | 2 (25) | 0.203 |  |
|  | - NSAIDs (incl. intolerance) | 11 (28.9) | 1 (12.5) | 0.660 |  |
|  | - Local anesthetics | 2 (5.3) | 0 (0) | 1.00 |  |
|  | - Other | 5 (13.2) | 2 (25) | 0.587 |  |
|  | Contrast agent (CT or MRT) | 2 (5.3) | 0 (0) | 1.00 |  |
|  | Unknown | 1 (2.7) | 0 (0) | 1.00 |  |
| Type-IV Sensitizations (n; % pos) |  | 4 (10.5) | 1 (12.5) | 1.00 |  |

Supplementary Table 5a – Female Cohort Anaphylaxis characteristics (TPSAB1 copy number increase positive vs. negative)
Statistical analysis was performed using a Mann-Whitney-Test (Kolmogorov-Smirnov rejects normal distribution)and fisher exact. Significant results were indicated with an asterisk (p<0.05*, p<0.01**). Anaphylaxis is graded according to Ring and Messmer^21^; (*) Grade of anaphylaxis was not available in 2 Individuals of the HaT positive group and 1 of the HaT negative group.
Abbreviations: IQR – interquartile range, HaT – hereditary alpha tryptasemia, pos – positive, AR – allergic rhinitis, NSAIDs – Nonsteroidal anti-inflammatory drugs, CT – computertomographie, MRT - magnetic resonance tomography.

|  |  |  | | |  |
| --- | --- | --- | --- | --- | --- |
|  |  | HaT Positive  n=6 | HaT Negative  n=19 | p = |  |
| Total IgE (median (IQR; U/ml) |  | 203 (484.9) | 60 (153.5) | 0.555 |  |
| AR (n; % pos) |  | 2 (33.3) | 1 (5.3) | 0.133 |  |
| History of anaphylaxis (n; % pos) (*) |  | 1 (16.7) | 18 (94.7) | <0.001** |  |
| Grade (n; % pos) | Anaphylaxis Grade I | 1 (16.7) | 0 (0) | 0.240 |  |
|  | Anaphylaxis Grade II | 0 (0) | 14 (73.7) | 0.003** |  |
|  | Anaphylaxis Grade III | 0 (0) | 2 (10.5) | 1.00 |  |
|  | Anaphylaxis Grade IV | 0 (0) | 0 (0) | # |  |
| Culprit (n; % pos) | Hymenoptera | 1 (16.7) | 18 (94.7) | <0.001** |  |
|  | Food | 0 (0) | 0 (0) | # |  |
|  | Medications total | 1 (16.7) | 1 (5.3) | 0.430 |  |
|  | - Antibiotics | 0 (0) | 0 (0) | # |  |
|  | - NSAIDs (incl. intolerance) | 0 (0) | 0 (0) | # |  |
|  | - Local anesthetics | 0 (0) | 0 (0) | # |  |
|  | - Other | 1 (16.7) | 3 (11.5) | 0.430 |  |
|  | Contrast agent (CT or MRT) | 0 (0) | 0 (0) | # |  |
|  | Unknown | 0 (0) | 0 (0) | # |  |
| Type-IV Sensitizations (n; % pos) |  | 0 (0) | 1 (5.3) | 1.00 |  |

Repository Table 5b – Male Cohort Anaphylaxis characteristics (TPSAB1 copy number increase positive vs. negative)
Statistical analysis was performed using a Mann-Whitney-Test (Kolmogorov-Smirnov rejects normal distribution) and fisher exact. Significant results were indicated with an asterisk (p<0.05*, p<0.01**). Anaphylaxis is graded according to Ring and Messmer^21^; (*) Grade of anaphylaxis was not available in 2 Individuals of the HaT negative group.
Abbreviations: IQR – interquartile range, HaT – hereditary alpha tryptasemia, pos – positive, AR – allergic rhinitis, NSAIDs – Nonsteroidal anti-inflammatory drugs, CT – computertomographie, MRT - magnetic resonance tomography.

|  |  | ***TPSAB1 copy number (HaT)*** | | |  |
| --- | --- | --- | --- | --- | --- |
|  |  | One additional copy / Duplication  n=37 | Two additional copies (a.o.Triplication)  n=7 | p = |  |
| Total IgE (median (IQR); U/ml) |  | 52 (189) | 33.5 (154.5) | 0.450 |  |
| AR (n; % pos) |  | 10 (27) | 3 (42.9) | 0.404 |  |
| History of anaphylaxis (n; % pos)(*) |  | 22 (59.5) | 2 (28.6) | 0.217 |  |
| Grade (n; % pos) | Anaphylaxis Grade I | 12 (32.4) | 0 (0) | 0.163 |  |
|  | Anaphylaxis Grade II | 4 (10.8) | 1 (14.3) | 1.00 |  |
|  | Anaphylaxis Grade III | 3 (8.1) | 1 (14.3) | 0.513 |  |
|  | Anaphylaxis Grade IV | 1 (2.7) | 0 (0) | 1.00 |  |
| Culprit (n; % pos) | Hymenoptera | 7 (18.9) | 2 (28.6) | 0.619 |  |
|  | Food | 4 (10.8) | 0 (0) | 1.00 |  |
|  | Medications total | 16 (43.2) | 2 (28.6) | 0.682 |  |
|  | - Antibiotics | 3 (8.1) | 0 (0) | 1.00 |  |
|  | - NSAIDs (incl. intolerance) | 10 (27) | 1 (14.3) | 0.659 |  |
|  | - Local anesthetics | 1 (2.7) | 1 (14.3) | 0.296 |  |
|  | - Other | 5 (13.5) | 1 (14.3) | 1.00 |  |
|  | Contrast agent (CT or MRT) | 2 (5.4) | 0 (0) | 1.00 |  |
|  | Unknown | 1 (2.7) | 0 (0) | 1.00 |  |
| Type-IV Sensitizations (n; % pos) |  | 4 (10.8) | 0 (0) | 1.00 |  |

Supplementary Table 6 – Anaphylaxis characteristics (Depending on additional TPSAB1 copy numbers)
Statistical analysis was performed using a t-test and fisher exact. Significant results were indicated with an asterisk (p<0,05*, p<0,01**). Anaphylaxis is graded according to Ring and Messmer^25^; (*) Grade of anaphylaxis was not available in 2 Individuals of the group with two additional copies.
Abbreviations: a.o. – among others, IQR – interquartile range, HaT – hereditary alpha tryptasemia, pos – positive, AR – allergic rhinitis, NSAIDs – Nonsteroidal anti-inflammatory drugs, CT – computertomographie, MRT - magnetic resonance tomography.
